# Device Management and Data Transport in IoT Networks Based on Visible Light Communication

**DOI:** 10.3390/s18082741

**Published:** 2018-08-20

**Authors:** Cheol-Min Kim, Seok-Joo Koh

**Affiliations:** School of Computer Science and Engineering, Kyungpook National University, Daegu 41566, Korea; cheolminkim@vanilet.pe.kr

**Keywords:** Visible Light Communication, Internet of Things, device management, data transport, experimentation, analysis

## Abstract

LED-based Visible Light Communication (VLC) has been proposed as the IEEE 802.15.7 standard and is regarded as a new wireless access medium in the Internet-of-Things (IoT) environment. With this trend, many works have already been made to improve the performance of VLC. However, the effectively integration of VLC services into IoT networks has not yet been sufficiently studied. In this paper, we propose a scheme for device management and data transport in IoT networks using VLC. Specifically, we discuss how to manage VLC transmitters and receivers, and to support VLC data transmission in IoT networks. The proposed scheme considers uni-directional VLC transmissions from transmitter to receivers for delivery of location-based VLC data. The backward transmission from VLC receivers will be made by using platform server and aggregation agents in the network. For validation and performance analysis, we implemented the proposed scheme with VLC-capable LED lights and open sources of oneM2M. From the experimental results for *virtual museum* services, we see that the VLC data packets can be exchanged within 590 ms, and the handover between VLC transmitters can be completed within 210 ms in the testbed network.

## 1. Introduction

Recently, Internet of Things (IoT) services have widely been used to improve our daily life. For example, with the help of IoT services, people can order what they need by pushing a button, or find the location where a fire has occurred by aggregating the measured values from sensors through the Internet.

For realizing the IoT, there has been lots of work undertaken on wireless communication technologies such as Bluetooth Low Energy (BLE) [1] and ZigBee [2], as well as the protocols to support packet delivery, such as Constrained Application Protocol (CoAP) [3], MQ Telemetry Transport (MQTT) [4], MQTT for Sensor Networks (MQTT-SN) [5], and so on. Moreover, there are also lots of standards and software to help develop IoT products, as shown by the Open Connectivity Foundation (OCF) [6], IoTivity [7], and oneM2M [8].

In the meantime, some industry areas may be subject to location-critical jobs and/or limited RF communication environments, such as huge factories, airplanes, underground facilities, and so on. Such environments need a specialized communication technology. Visible Light Communication (VLC), which is defined in the IEEE 802.15.7, is one of the communication solutions for meeting these characteristics [9]. VLC communication is carried out between transmitter and receivers using visible light. Based on this characteristic, VLC offers no interference to existing RF-based communications. Also, it does not require a license to use the spectrum of visible light. Moreover, VLC provides accurate location-based communication, in contrast to existing low-power communication, such as BLE and Near Field Communication (NFC) [10].

Nowadays, LED lights are widely spread in our daily life. A great deal of work has been done on how to improve the performance of LED-based VLC in the physical and MAC layers [11,12,13,14]. The IEEE 802.15.7 group has recently standardized Optical Wireless Communications (OWC) [9]. However, how to effectively integrate VLC services into the IoT network has not yet been studied enough. In this paper, we propose a scheme for device management and data transport in IoT networks using VLC. Specifically, we discuss how to manage VLC devices (VLC transmitters and receivers) and support the VLC data transmission in the IoT networks based on the oneM2M standard. In the proposed VLC-based IoT scheme, we consider the uni-directional VLC transmissions from VLC transmitter to VLC receivers for location-based VLC data service. The backward transmission from VLC receivers to VLC transmitter will be made using the IoT platform server and aggregation agents in the IoT network. By implementation and testbed experimentation, we will also perform the validation and performance analysis for the proposed scheme.

This paper is organized as follows. Section 2 briefly describes the related works on VLC and IoT. In Section 3, we propose the reference network model for the VLC-based IoT scheme and the device management and VLC data transport operations in the IoT networks. In Section 4, we discuss implementation and experimentation, with a performance analysis for the proposed scheme. In Section 5, we conclude this paper and describe future works.

## 2. Related Work on VLC and IoT

The VLC standard was developed by IEEE 802.15 Task Group 7 in 2011 [9]. The VLC standard defines the PHY and MAC layers for short-range optical wireless communication using visible light. The specification classifies devices as infrastructure, mobile, and vehicle, and defines the MAC topologies: peer-to-peer, star, and broadcast. The infrastructure device is a fixed coordinator and is unconstrained, and has a sufficient power source. The mobile device is a constrained device which has a limited power supply, a weak light source, and a short visibility range. The vehicle device is an unconstrained device that has a moderate power supply, intense light source, and long visibility range. In the peer-to-peer and start topology, there is a coordinator device, which acts as controller. The controller is set by the administrator or association algorithms. Generally, the coordinator will be the powered devices. The IEEE 802.15.7 task group is now developing the revised version of the VLC standard, called IEEE 802.15.7r1. This standard includes support for infrared and near ultraviolet wavelengths as additional light sources. Also, it supports Optical Camera Communications (OCC) and LiFi. OCC can be used for devices with image sensors, such as cameras and smartphones, to act as VLC receivers. Currently, the IEEE 802.15.7 revision work is being progressed as IEEE 802.15.7m. This standard is the same as IEEE 802.15.7r1, except without support for LiFi [15,16,17,18].

The IPv6 over Low-power Wireless Personal Area Networks (6LoWPAN) was made to support IP-based communication over the IEEE 802.15.4 Wireless Personal Area Network (WPAN) in the Internet Engineering Task Force (IETF) [19,20]. The packet size in IEEE 802.15.4 is 127 octets at maximum, whereas an IPv6 packet needs at least 1280 octets of Maximum Transmission Unit (MTU). Thus, the 6LoWPAN defines a new adaptation layer to support IPv6 packets. This adaptation layer defines multiple frame formats with compressed IPv6 headers to support IPv6 communication in IEEE 802.15.4 networks [21]. This has now evolved to support BLE and NFC [22,23].

The Constrained Application Protocol (CoAP) was developed for constrained or sensor nodes by the IETF Constrained RESTful Environments (CoRE) Working Group (WG) [3]. CoAP is derived from the HyperText Transfer Protocol (HTTP), including the server/client model and request/response operations. Constrained devices have difficulty using the original HTTP, because their physical and computing resources are limited. Therefore, CoAP was designed to have very low overhead by adopting the User Datagram Protocol (UDP) and a binary-based message format. The CoAP has two message types: confirmable (CON) and non-confirmable (NON). A CON message is used for providing reliable message transfer. Every CON message must be acknowledged by ACK message. The IETF CoRE WG is now developing group communication; block-wise transfers; CoAP over TCP, TLS, and WebSockets; and publish-subscribe broker and congestion control [24,25,26,27,28].

The MQ Telemetry Transport (MQTT) [4] is based on the TCP-based publisher/subscriber model using a messaging protocol. It is also designed to transport messages between constrained devices. MQTT operates with a topic. One device subscribes to the topic, and another device publishes data to the topic. When a publisher publishes messages to a topic, the server forwards the topic messages to the subscribers. Nowadays, a simpler MQTT protocol, called MQTT for Sensor Networks (MQTT-SN), is being developed to support more constrained devices, such as sensor devices [5]. The MQTT-SN is interoperable with the MQTT protocol though the MQTT-SN gateway.

oneM2M is a global organization for standard development and efficient deployment of M2M communications systems [8]. Until now, oneM2M has released two versions of the M2M standard: releases 1 and 2. Release 1 includes the common service platform for IoT, which defines the Common Services Function (CSF) for data saving/sharing, device management, group management, subscription and notification, location, billing, and so on. This release also contains fundamental security features such as authentication and access control. In addition, it defines message primitives and protocol bindings. Release 2 includes the interoperation with AllJoyn, OCF, Lightweight M2M (LWM2M), 3GPP Rel-13. It also includes various features needed by industries.

As described previously, much work has been done on how to improve the performance of VLC transmission and on the protocols and platforms for IoT, such as 6LoWPAN, CoAP, MQTT, and oneM2M. However, how to effectively integrate VLC services into IoT networks has not been studied enough. That is, VLC has not been considered as a wireless access medium for IoT. Thus, in this paper, we propose a framework for VLC-based IoT networks and also discuss the operations for device management, data transport and handover in IoT networks using VLC.

## 3. Proposed VLC-Based IoT Scheme

### 3.1. Network Reference Model

**Figure 1:**
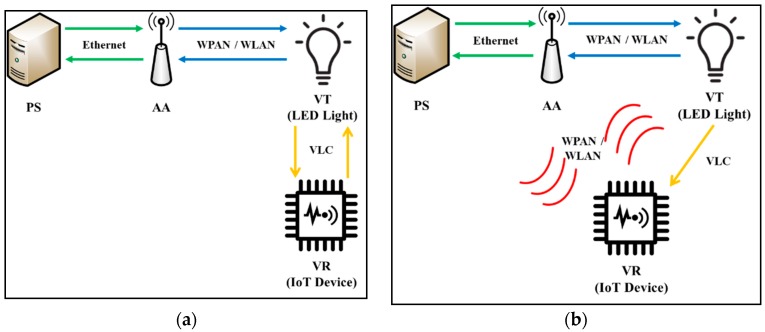
VLC communication scenarios: (**a**) bi-directional VLC; (**b**) uni-directional VLC.

For VLC-based IoT networks, we consider the following four types of network nodes: Platform Server (PS), Aggregation Agent (AA), VLC Transmitter (VT), and VLC Receiver (VR). There are two possible communication scenarios between VT and VR, as shown in Figure 1. Figure 1a depicts bi-directional VLC between VT and VR, in which both uplink and downlink VLC transmissions are allowed. On the other hand, Figure 1b shows uni-directional VLC from VT to VR, in which only downlink VLC transmission is allowed from VT to VR, and the uplink or backward transmission will be made between VR and AA by using another network link, such as WLAN or WPAN.

It is noted that most real-world VLC deployments follow the uni-directional model displayed in Figure 1b, even though the IEEE 802.15.7 specification defines both bi-directional and uni-directional models. This is because the implementation of bi-directional VLC requires multiple light sources or more complex MAC algorithms. Based on this observation, this paper proposes the VLC-based IoT scheme based on the uni-directional VLC model. The bi-directional VLC model will be considered for further study.

**Figure 2:**
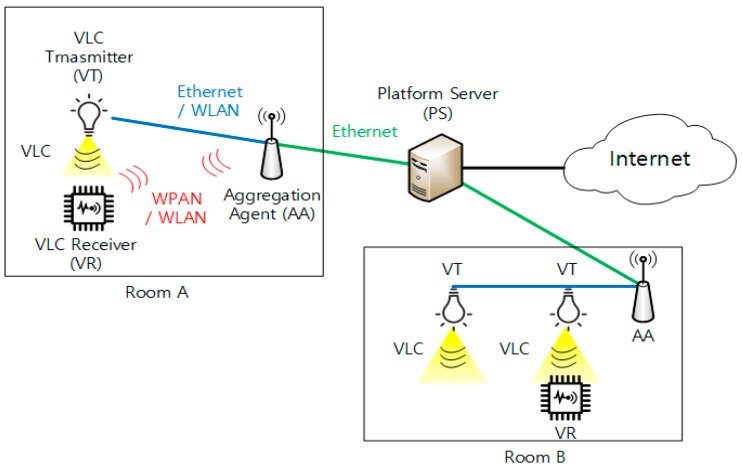
Network reference model for VLC-based IoT.

Figure 2 shows the network reference model for the VLC-based IoT scheme, in which the network entities are classified as Platform Server (PS), Aggregation Agent (AA), VLC Transmitter (VT), and VLC Receiver (VR). Each network entity provides the following functionality:

- Platform Server (PS)
  PS performs overall management for all devices, including AAs, VTs, and VRs, in the VLC-based IoT network through the operations of device initialization, monitoring, and VR handover. PS also controls location-specific VLC data (from VT to VR) and service-specific data (between AA and VR) by using the data transport operation. PS is connected to the Internet.
- Aggregation Agent (AA)
  AA manages VT and VR devices locally. It keeps and updates the VT–VR mapping information for its attached VT through the device initialization, monitoring, and VR handover operations. That is, AA can realize the list of VRs that is connected to a specific VT. AA also supports the VR service channel with VR, in which AA exchanges service data with VR. AA is also used to relay location-specific VLC data between PS and VT, and service-specific data between VR and PS.
- VLC Transmitter (VT)
  VT controls the LED light. When VT is connected and registered to AA, it transmits location-specific data with VLC frames toward VRs.
- VLC Receiver (VR)
  VR is a user or sensor device which acts as a VLC receiver. When VR receives VLC frames with the location-specific data from VT, it will begin the registration with AA. If VR is connected to AA, it may request service data from AA, based on the context of the location-specific data.

In Figure 2, there are four types of network links. The green line linking AA and PS could possibly be Ethernet. The blue line between AA and PS could be Ethernet or Wireless LAN (WLAN) [29]. The yellow triangle illustrates the VLC frames transmitted by LED light. The red links indicate the service channel between AA and VR. This service channel could be WLAN or WPAN, such as ZigBee, BLE, Z-Wave, etc. It is noted in the proposed scheme that this service channel is employed to provide the uplink or backward communication from VR to AA or PS, as discussed with respect to Figure 1b. 

### 3.2. Protocol Stacks Based on Uni-Directional VLC

**Figure 3:**
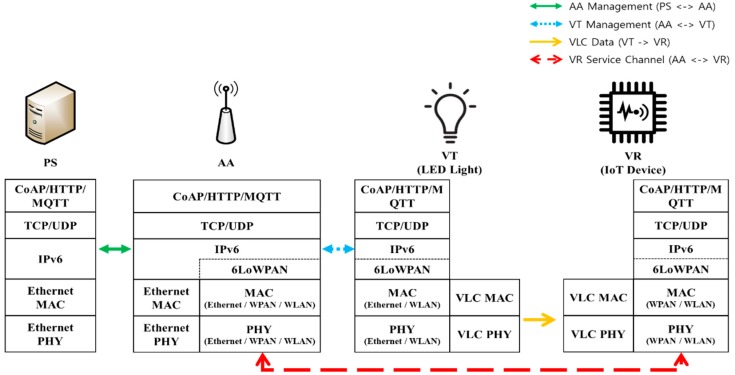
Protocol stacks used for VLC-based IoT.

Figure 3 shows the protocol stack used by each network entity in the proposed VLC-based IoT scheme, which is based on IPv6 networks.

In the figure, PS and AA are connected through Ethernet. Connections between AA and VT can be established through Ethernet, WLAN, or WPAN. 6LoWPAN may be used between AA and VT, depending on the underlying link, such as BLE or ZigBee. Each VT is connected to an AA. PS can send location-specific VLC data to each VT via AA. 

In the meantime, VR receives the VLC data frames from VT. This VLC data frame can contain location-specific data given by PS. Based on this location-specific VLC data, VR establishes the VR service channel with AA by using WPAN or WLAN. To further discuss the use of location-specific VLC data and the VR service channel, let us take an example of the *museum* service, which consists of a single PS (acting as a museum administrator) and many AAs (an AA may be established at each floor in the museum). It is assumed that each AA is responsible for many VTs, and each VT supports a particular item in the museum (e.g., a famous picture). A visitor (user) is equipped with a VR device and moves across VTs to see different items. In this example, PS can provide each VT with location-specific data (an item identifier), and when VR visits the VT, that location-specific information (item identifier) is delivered from VT to VR via the VLC transmission. Based on the item identifier, VR will establish the service channel with AA and download the item (picture) file from the AA. In this way, the location-specific VLC data (possibly initiated by PS and/or AA) is delivered from VT to VR via the uni-directional VLC transmission, and the VR service channel is used between VR and AA for uplink or backward data delivery. This is an example for VLC-based IoT service, and its extension could be possible, depending on the status of VLC deployment and the features of target VLC services.

### 3.3. Device Management and Data Transport Operations

In this section, we describe the VLC-based IoT operations, which are divided into device initialization and monitoring, VLC data transport, and VR handover operations. 

#### 3.3.1. Device Initialization

**Figure 4:**
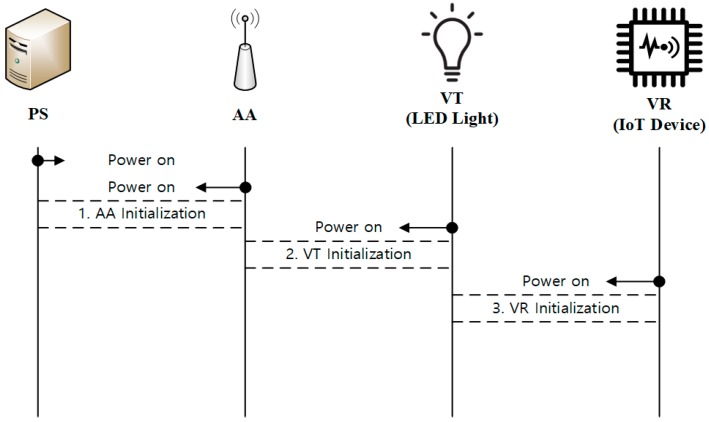
Overview of device initialization.

During device initialization, all of the devices (AA, VT, and VR) are registered with PS. Figure 4 shows the overall steps for device initialization.

**Figure 5:**
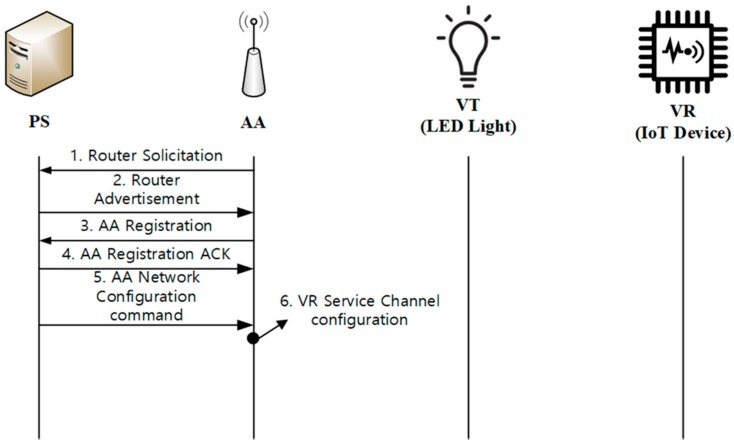
AA initialization.

Figure 5 shows the AA initialization process between PS and AA, which is divided into the IP connection establishment and the AA registration to PS. When an AA turns on, it finds the PS by sending a Router Solicitation message, as done in a typical IP network. Then, PS responds to AA with a Router Advertisement. The Router Advertisement message contains the IP address of PS, including information related to the Domain Name System (DNS) server and the Dynamic Host Configuration Protocol (DHCP) server, if available. In certain cases, PS may send the Router Advertisement messages periodically. AA configures an IPv6 address after receiving the Router Advertisement message. When the address configuration is completed, AA sends the AA Registration message to PS. After the processing of appropriate operations based on the AA Registration, PS will reply with the AA Registration ACK message. If necessary, PS may send the AA Network Configuration commands to the AA so as to provide the information required for configuration of VR Service Channel at AA. Processes 3 to 6 are accomplished at the application level.

**Figure 6:**
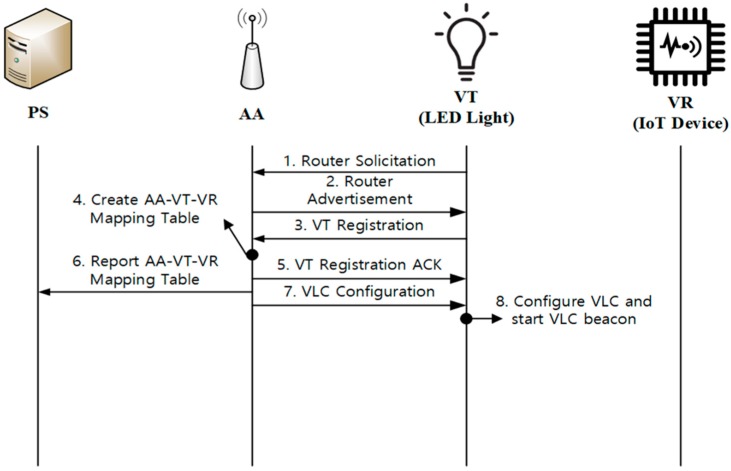
VT initialization.

Figure 6 shows the VT initialization process, which is divided into IP connection establishment and VT registration to AA. Because VT is a lighting device with VLC functionality, when VT is powered on, it operates as a normal light bulb. It is noted that our scheme could also be used for control of a light bulb, such as switching the light on or off or changing dimming rates. After being powered on, VT is connected to an AA by sending a Router Solicitation message and receiving a Router Advertisement message from AA. When VT receives a Router Advertisement message, it configures an IPv6 address to communicate with AA. After that, VT sends the VT Registration message to AA, which may contain the VT identifier and status information on the attached LED light. For VLC device management, AA needs to manage the mapping information on its downstream VTs and VRs. For this purpose, AA creates and maintains an AA-VT-VR mapping table, and it will report this mapping information to PS. Now, AA responds to VT with the VT Registration ACK message. If necessary, AA sends the VLC Configuration message to the registered VT, which contains the parameters required for VLC configuration. Then, the VT configures its VLC function and starts to transmit VLC beacons toward the promising VRs. It is noted that processes 3 to 8 are done at the application level.

**Figure 7:**
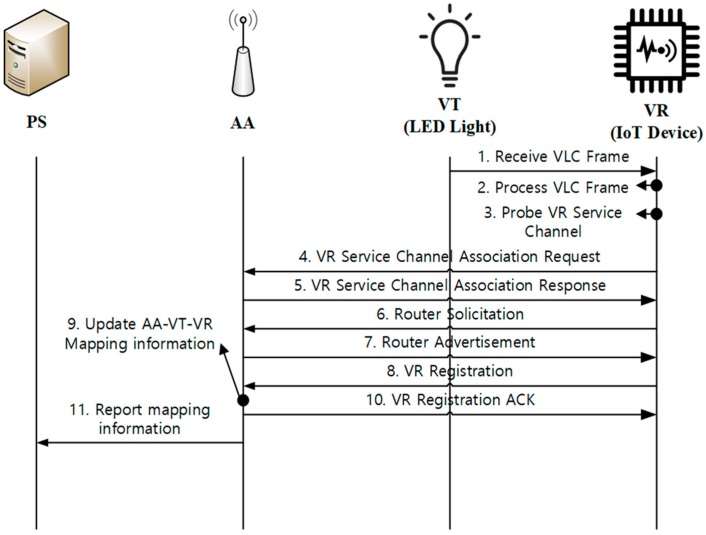
VR initialization.

**Figure 8:**
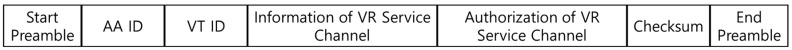
VLC frame format.

Figure 7 shows the VR initialization process. When VR is turned on, it waits to receive any VLC frames. Once a VLC Frame is received, VR decodes the VLC frame and tries to establish the VR Service Channel with the associated AA. After associating VR Service Channel, VR configures an IPv6 address to communicate with AA, exchanging Router Solicitation and Router Advertisement messages. When an IPv6 address is configured, VR tries to register with AA by sending VR Registration message. AA updates the AA-VT-VR mapping table created before and replies to VR with a VR Registration ACK message. After VR registration is completed, AA reports the new VR to PS by sending the mapping table. Steps 3 to 8 are done at the application level. Figure 8 gives an example of the VLC format and how fields in the VLC frame are used for the VR initialization operation. The VLC frame contains the *start* and *end preamble* for distinguishing VLC frames. *AA ID* and *VT ID* will be used for VR registration, device monitoring, and VLC data transport. In addition, the *information on VR Service Channel* can be defined. For example, when WLAN is used between VR and AA, the Basic Service Set Identifier (BSSID) of Access Point (AP) for AA may be included as the *information of VR Service Channel*. The *authorization of VR Service Channel* may be added when access control from unauthorized requests is needed. Both fields are related to associating VR Service Channel with AA. A *checksum* field may be included for checking the validity of the received VLC frame.

#### 3.3.2. Device Monitoring

**Figure 9:**
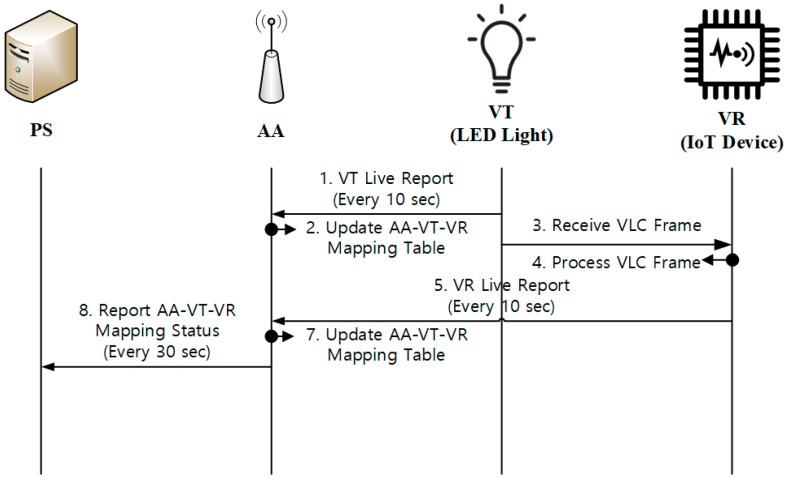
Device monitoring.

Device monitoring is used for PS to keep track of the status of the AA, VT, and VR devices in the network. Figure 9 shows the device monitoring operations, in which the VT and VR devices periodically report the status to AA, and AA sends aggregate status information to PS.

As shown in the figure, AA always keep the AA-VT-VR mapping table for device monitoring. VT and VR may use a timer for periodic reporting (e.g., every 10 s). Such a report message contains the identifiers of the concerned VT and VR. On reception of the reports from VT and VR, AA will update its AA-VT-VR mapping table. Then, AA also reports the aggregate status information to PS periodically (e.g., every 30 s). It is noted that an appropriate timer value may be configured, depending on the network deployment and the service features to be considered.

#### 3.3.3. VLC Data Transport

In the proposed scheme, the VR data is classified into the following two types. One is location-specific data, which is contained in the VLC frames that VT transmits to VRs. The other one is service data, which is delivered through the VR service channel between AA and VR.

**Figure 10:**
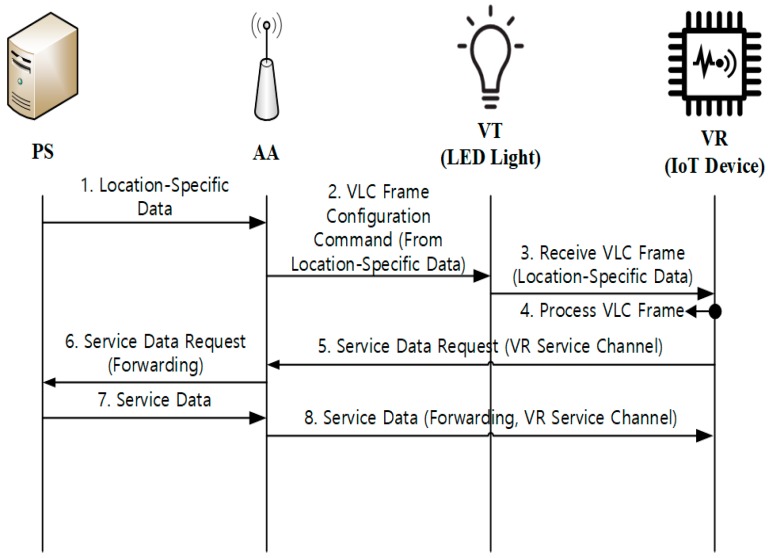
VLC data transport.

Figure 10 shows the data transport operations, in which VR receives location-specific data from VT and service-specific data is exchanged between AA and VR. For the example of the *museum* service, the VR device may be used to give detailed information on the concerned exhibition item (e.g., description on the item) to the visiting user. In this case, the location-specific data contains the IDs of AA and VT, the channel number of the target item, etc. Please note that such information is specific to a particular VT or item. The VR device might be a smartphone with a mobile guide application for the museum, or a dedicated device that is given by the museum. Based on the location-specific data, VR will try to get more detailed and additional information on the target item from AA, which is served by the service data of VR Service Channel.

#### 3.3.4. VR Handover

**Figure 11:**
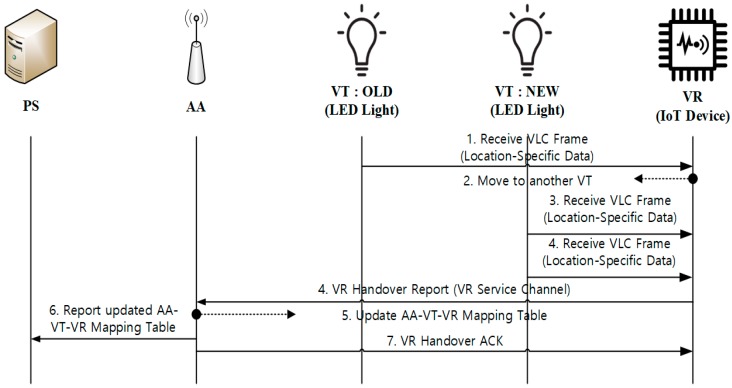
VR handover across VTs.

If VR is a mobile device, it may move around and across different VTs in the network. When a VR moves to another VT, VR performs the handover operation, as described in Figure 11. VR will detect a handover event if it receives a new VLC frame from a different VT. When the handover event is detected, VR sends a VR Handover Report message to AA. The message contains the identifiers of old and new VTs. If AA receives the handover report, it will update the AA-VT-VR mapping table, and send a report to PS. After that, AA responds to VR with a VR Handover ACK message through the VR Service Channel.

## 4. Experimentation and Discussion

For validation and evaluation, we implemented the proposed VLC-based IoT scheme by using the oneM2M-based IoT platform and performed testbed experimentation for a simple VLC service, *virtual museum*.

### 4.1. Testbed Configuration

**Figure 12:**
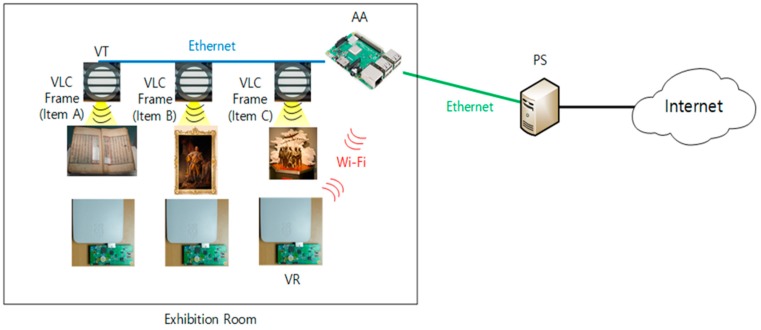
Testbed network configuration for *virtual museum* service.

In the *virtual museum* service scenario, there are lots of exhibition rooms. Each room has multiple exhibition items, each of which is associated with a VT. Figure 12 shows the network configuration for the *virtual museum*, in which AA is located at the exhibition room and connected to PS. Multiple VTs are connected to AA through Ethernet in the room. The PS is connected to Internet for museum administrator. A museum visitor (user), who is equipped with VR, will move across different VTs in the room. The VR Service Channel between VR and AA is based on Wi-Fi connection.

For experimentation, we implemented the proposed scheme by using oneM2M-based platform software, called the *Mobius* platform [30], which is based on the standard of oneM2M release 1. In the Mobius platform, the *Mobius server* for PS is implemented as *Infrastructure Node Common Services Entity (IN-CSE)* in oneM2M [31], the *nCube-Rosemary* for AA is implemented as *Middle Node of Common Services Entity (MN-CSE)* in oneM2M [32], and *nCube-Thyme* is used for implementation of VT and VR as *Application Dedicated Node of Application Entity (ADN-AE)* in oneM2M [33]. The *Mobius server* for PS is installed on a desktop PC with *Ubuntu 16.04.1 LTS*, and the *nCube-Rosemary* for AA is installed on a *Raspberry Pi* with *Raspbian OS*. VT is also installed on *Raspberry Pi* with *nCube-Thyme* and a VLC-capable commercial LED light. Another *Raspberry Pi* is used for VR with Linux OS, VLC receiver, and *nCube-Thyme*. PS is equipped with its own DHCPv6 server, which is implemented using the *dnsmasq* package from the *Ubuntu* repository. AA also has its own DHCPv6 server, and the functionality of Access Point (AP) is implemented by the *hostapd* package from the *Raspbian* repository.

**Figure 13:**
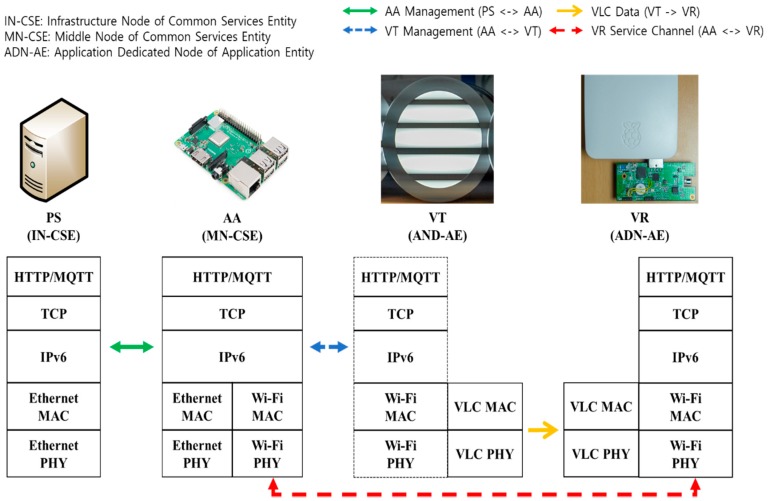
Protocol stack for implementation of VLC-based IoT.

Figure 13 illustrates the protocol stacks used in implementation and experimentation. PS and AA are connected through Ethernet. The current version of VT is a *Field Programmable Gate Array (FPGA)*-controlled commercial LED light and a pre-installed firmware for VLC function. An enhanced version of VT will be considered for further study. The VR is attached to the FPGA-controlled Photo Diode (PD) for functionality as a VLC receiver. The VR receives the VLC data through *Universal Asynchronous Receiver/Transmitter (UART)* communication with the VLC receiver device. The VR service channel between AA and VR is established by using Wi-Fi.

**Figure 14:**
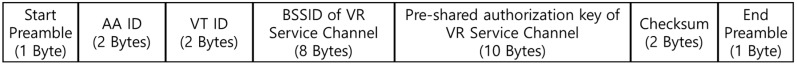
VLC frame structure used in experimentation.

For VLC communication, VT and VR use *Pulse Width Modulation (PWM)*-based modulation. The black horizontal lines of VT, as shown in Figure 12 and Figure 13, imply that the VLC frames are transmitted through visible light. The PD-based VLC receiver detects the VLC frames from the received light at PD. The structure of a VLC frame used in the experiment is depicted in Figure 14. In experimentation, we employed 6 VTs, and each VT has a unique VT ID.

### 4.2. Experimentation Results

#### 4.2.1. Device Initialization

**Figure 15:**
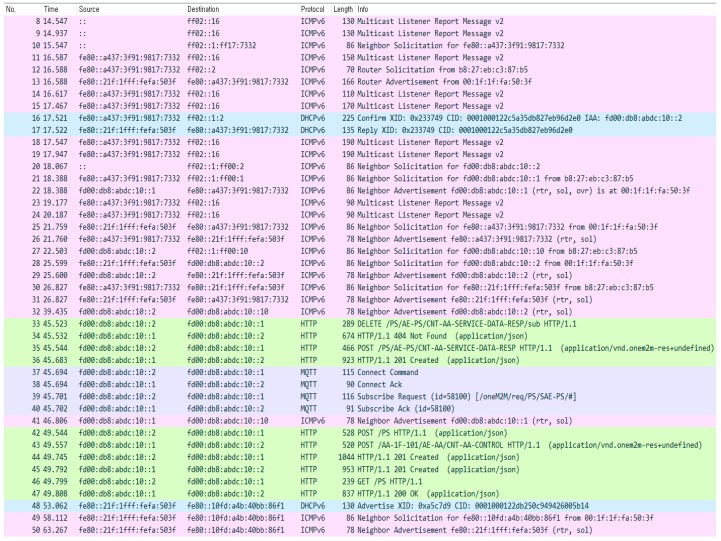
Packets captured during AA initialization.

Figure 15 shows the packet capturing results for the AA initialization process, as shown in Figure 5. In this experiment, PS is equipped with a local DHCPv6 server so that AA gets an IPv6 address (see the packets no. 13~17). After the configuration of the IPv6 address, AA creates and subscribes to a container named *CNT-AA-SERVICE-DATA-RESP*, which is used for receiving service data from PS during data transport operation (see the packets no. 33~40). Then, AA registers to PS by exchanging Common Service Entity (CSE) information (see packets no. 42, 44, 46, and 47). After registration of AA to PS, PS sends the AA Network Configuration message to the container (*CNT-AA-CONTROL*) of AA (see packets no. 43 and 45).

**Figure 16:**
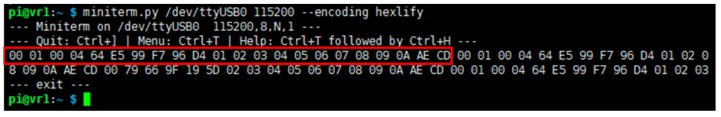
Example of VLC frame used in experimentation.

The current version of VT is installed as pre-installed firmware of VLC functions without IP communication. Therefore, the location-specific data is included in the firmware. An enhanced version of VT will be made for further study, which will act as an ADN-AE device of a oneM2M platform. Figure 16 shows the information contained in a VLC frame transmitted by VT, following the VLC frame format in Figure 14.

**Figure 17:**
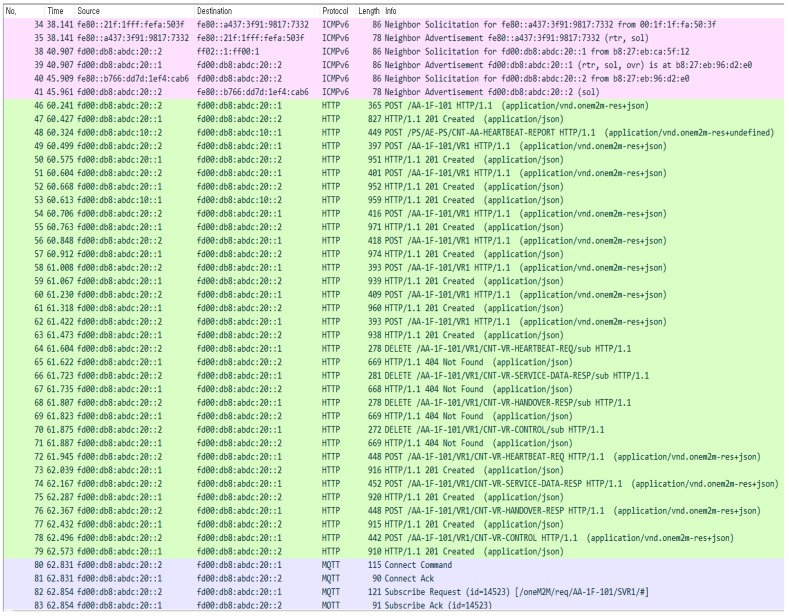
Packets captured during VR Initialization.

Figure 17 shows the packets captured during VR initialization. In the figure, VR finds a local router (AA) and configures its IPv6 address. AA has its own local DHCPv6 server. After configuring an IPv6 address, VR is registered to AA. Because VR is an ADN-AE device, all of the information on oneM2M should be recorded into a local CSE. Therefore, VR creates AE and a container, and subscribes to AA (see the packets no. from 46 to 83), in which AE creation is shown in packets 46 to 47, the container is from 48 to 63, and the subscription is from 64 to 83. The subscription container named *CNT-VR-HEARTBEAT-*REQ is for device monitoring, *CNT-VR-SERVICE-DATA-*RESP is for receiving service data during data transport operation, and *CNT-VR-HANDOVER-*RESP is for support VR handover.

#### 4.2.2. Device Monitoring

**Figure 18:**
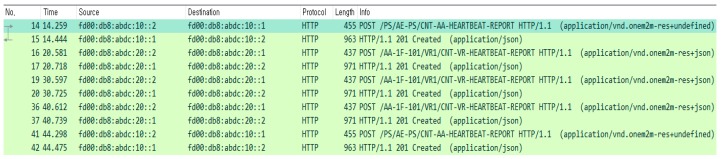
Packets captured during device monitoring.

As mentioned earlier, we perform the device monitoring operation periodically. Figure 18 shows the packet capturing results during device monitoring. Every node has a local timer to report the current mapping status. In the figure, VR sends the reports to AA for every 10 s (see packets of 16, 19, and 36), and AA reports the current mapping table to PS for every 30 s (in packets of 14 and 41). 

#### 4.2.3. VLC Data Transport

**Figure 19:**
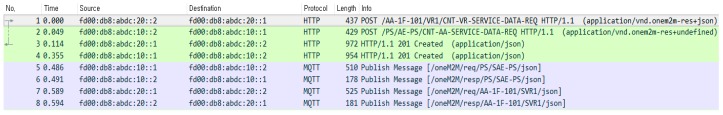
Packets captured during the VLC Data Transport operation.

In experimentation, VR is an ADN-AE device, and VT uses uni-directional broadcast VLC. Figure 19 shows the captured packets during the data transport operation. In the figure, VR requests service data from PS and AA through the VR Service Channel. The service data request (of packet no. 1) contains the identifier of VT. When AA receives this request, it forwards the service data request to PS (packet no. 2). Then, PS prepares service data and publishes service data to AA (packet no. 5). Finally, AA forwards the service data to VR (packets no. 7 and 8).

#### 4.2.4. VR Handover

**Figure 20:**
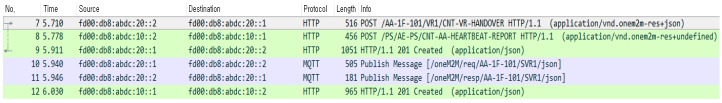
Packets captured during VR Handover operation.

Figure 20 shows the flow of packets during VR handover. When VR moves to another VT and detects a handover, it sends a handover notification message to AA (packet no. 7). The message contains identifiers of old and new VTs. AA updates its mapping table and reports the updated table to PS (see the packets no. 8, 12). Then, AA publishes a handover completion message to VR (packet no. 10).

### 4.3. Performance Analysis

**Figure 21:**
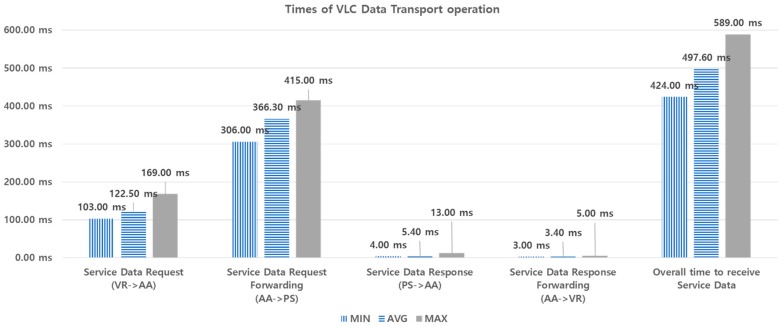
Times taken during VLC data transport.

**Figure 22:**
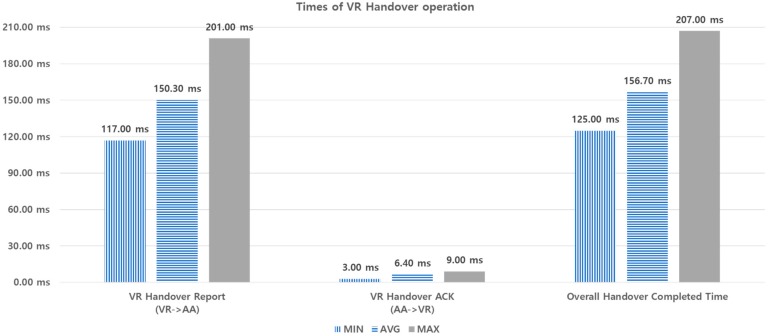
Times taken during VR handover.

For performance analysis by experimentation, we measured the times required for the VLC data transport and VR handover operations. For each performance metric, 10 experiments are made in the same way, and the min., max., and average values are obtained, as shown in Figure 21 and Figure 22.

Figure 21 shows the times taken during VLC Data Transport operation. In the figure, the service data request from VR to AA takes 169 ms, maximum. The forwarding of the service data request from AA to PS takes 415 ms, maximum. Such delay includes the time required to make *contentInstance* of each request. The service data response from PS to AA takes 13 ms, maximum, and service data response forwarding from AA to VR takes 5 ms, maximum. These delays are relatively small, compared to the previous two messages. This is because these two messages do not create *contentInstance*. The overall delay in receiving the service data is a maximum of 589 ms. In this experiment, we used a tiny piece of data. It may take a longer time if larger data is used.

Figure 22 shows the times taken during the VR handover operation. The VR handover report from VR to AA takes 201 ms, maximum. This operation includes the time to create a HTTP message from VR to AA, and vice versa, and the *contentInstance* of AA. On the other hand, the VR handover ACK from AA to VR takes 9 ms, maximum. The ACK operation does not create *contentInstance*. The overall handover delay for handover completion is 207 ms, maximum. From the figure, we can also see that the maximum value of handover delay will not exceed 207 ms, because it does not need data.

### 4.4. Discussion

The *virtual museum* service in the experiment is simulated under a testbed environment, in which we performed the experimentations with several real VLC transmitters and receivers. The VLC transmitters used in the experiment are 18 W, white-colored (5700 K), 120 degrees of view angle, 1440 lm flux LED lights. The VLC modulation uses PWM with a 1.6 ms period and 4 m communication range. The PD-based VLC receiver has a 115,200 baud rate through UART.

To extend the *virtual museum* service into real-world environments, we need to consider large numbers of VLC transmitters and receivers, since there are many exhibition rooms and a lot of users in the museum. In this case, the performance may depend on how to effectively configure the platform server and many aggregation agents with VLC transmitters and receivers in the network. In addition, the security issue should be considered, because the VLC frames have no encryption scheme and contain channel information of VR service network. In the deployment of VLC transmitters in real networks, we also need to consider that VLC frames are generated with the same modulation scheme and wavelength, and thus this may cause interference.

## 5. Conclusions and Future Work

In this paper, we designed and proposed a device management and data transport scheme for IoT networks based on VLC. For validation and performance analysis, we implemented the proposed VLC-based IoT scheme with VLC-capable LED lights and the open-source oneM2M platform. We also conducted testbed experimentation for *virtual museum* services. From the experimental results, we see that the VLC data packets can be exchanged within 590 ms, and the handover between VLC transmitters can be completed within around 210 ms in the testbed network.

To the best of our knowledge, this paper is the first proposal for VLC-based IoT. In further works, we plan to implement and investigate the proposed scheme with a protocol stack based on CoAP/UDP/6LoWPAN. We will also consider the bi-directional VLC communication scenario in VLC-based IoT networks. In addition, we will include a comparison of the proposed VLC-based IoT scheme with existing IoT schemes which use other access network technologies, such as Bluetooth and ZigBee, and with other candidate schemes for VLC-based IoT. The testbed experimentation and performance comparisons will be made for a variety of network environments.
